# Microsphere—Toward Future of Optical Microscopes

**DOI:** 10.1016/j.isci.2020.101211

**Published:** 2020-05-28

**Authors:** Lianwei Chen, Yan Zhou, Rui Zhou, Minghui Hong

**Affiliations:** 1 Department of Electrical and Computer Engineering, National University of Singapore, 4 Engineering Drive 3, Singapore 117576, Singapore; 2 NUS Graduate School for Integrative Sciences and Engineering, National University of Singapore, 21 Lower Kent Ridge, Singapore 119077, Singapore; 3 School of Aerospace Engineering, Xiamen University, Xiamen 361102, China

**Keywords:** Optical Imaging, Photonics, Nanotechnology

## Abstract

Optical microscope is one of the most widely used imaging tools for its great flexibility, reliable design, and low cost. Optical microsphere nanoscope (OMN) is invented as a method that can greatly enhance the observation power of conventional optical microscopes. In this perspective, the promising outlook for this approach is briefly discussed. There exists a great freedom to apply this method in various applications. OMN has been successfully commercialized. Our past experience and strategies are summarized in this perspective, which serves as a good reference for the future technology entrepreneurs. Based on our story and model, the factors for success are listed. It can be used to evaluate other commercialization projects and find out the directions that require further improvement.

## Optical Microscope and Microsphere

### Challenges for Optical Microscopes

The first scientific report on the function of the spherical lens was in the works of Seneca (4 BC–AD 65), which described the function of transparent spheres to produce a magnified image. The continuous development of optics then led to the invention of optical microscopes, which opened the gate for many scientific discoveries (Croft, 2006, Lloyd and Jefferis, 1995, Max and Emil, 1975). However, the optical microscope was soon found to be restricted by a fundamental limitation, namely, the diffraction limit (Abbe, 1873, Rayleigh, 1879). Diffraction limit originated from the wave nature of light. For the objects smaller than the diffraction limit, optical microscopes can hardly resolve it. Diffraction limit sets the boundary for the resolution. For commercial systems, ~200 nm resolution becomes challenging for conventional visible spectrum optical microscopes functioning in ambient air. Compared with it, other nano-imaging tools, such as scanning electron microscopy (SEM) and tunneling electron microscopy (TEM), can achieve resolution down to atomic level.

However, optical microscopes have unrivaled advantages and the following unique features. (1) Optical microscopes are not limited by their working environments. They can function well in air, water, and oil environments. Conventional SEM and TEM need to be operated in vacuum. Many biological samples, such as living cells, cannot be directly characterized under vacuum, where special pre-treatments are required. The optical microsphere nanoscope (OMN) can form the image of the target sample immediately, whereas the electron-based microscopy relies on a scanning operation. Efficiency is critical for many applications such as defects detection of a product on the streamline, and OMN is favored from this perspective. Furthermore, although great progresses have been made on characterization of non-conductive samples, there still exist limitations and the point-to-point scanning manner also limits the operation efficiency and flexibility. Hence, optical microscopes are more flexible and suitable for a broad range of samples. (2) Optical microscopes provide dynamic and real-time imaging, where the operation does not require scanning. It can be used to capture videos and monitor the details in dynamic or ultra-fast processes, useful for the *in situ* analyses in chemical reactions. (3) The information available in the optical microscope is abundant. It characterizes the not only morphology and shapes but also many light properties such as spectrum, polarization, and phase, which could reveal extra information of the samples of interest. (4) Most optical microscopes do not require complicated sample preparation. It is versatile and cost efficient for many large-scale applications, such as quality control and defects detection in manufacturing lines. It also does not introduce any contamination into the sample. (5) Optical microscope is a good platform to be further upgraded for different purposes. Fluorescent dyes, Raman spectroscopy, and many other technologies can be integrated into an optical microscope. It can also be applied in fabrication systems, such as 3D printing and optical lithography to provide process monitoring in real time. Owing to these features, the optical microscope stands firmly as a widely used instrument in both research laboratories and industries. For the samples with feature sizes larger than the diffraction limit, the optical microscope remains to be the first choice. At present, the bottleneck challenge for the optical microscopes is its capability of imaging resolution. This fundamental limitation has existed for so long, and many people had even believed that it was impossible to breakthrough such physical law. Researchers have been seeking alternative solutions to bypass it.

### Microsphere Creating a New Route

**Figure 1:**
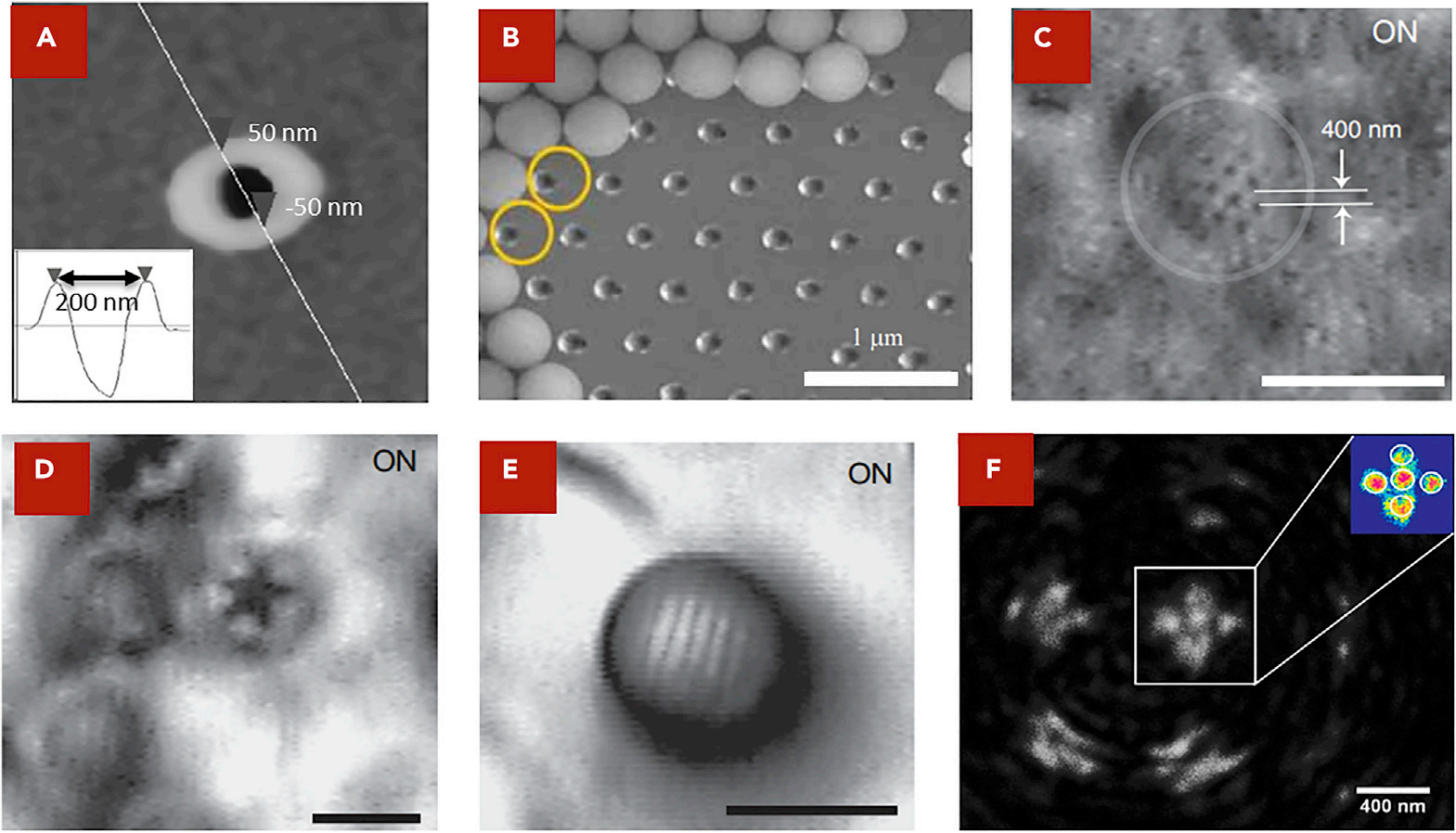
Imaging Results for Optical Microsphere Nanoscope (A) Scanning electron microscope (SEM) characterization to demonstrate the generation of the holes with sizes smaller than the diffraction limit. The sizes of the holes were measured after the laser cleaning experiment, with a full width at half maximum of ~100 nm (bottom-left inset showing the result of a surface profile measurement). (B) SEM to characterize the position of the holes in an off-axis illumination condition. The original positions of the microspheres are marked by yellow spheres. A GST film was used as the substrate, and holes were fabricated with a 30° off-axis illumination respect to the normal incidence (scale bar, 1 μm). (C–E) Experimental results to demonstrate microsphere imaging in the contact mode. (C) A gold-coated anodic aluminum oxide (AAO) sample was used as the target object. The smallest features on this sample were nano-pores with 50 nm diameter spaced 50 nm apart. Microspheres with a diameter of 4.74 μm were deposited on the surface. One of them was marked by white dashed lines to indicate its borders. In the image, the gap between the nano-holes was measured to be 400 nm with an 8× magnification factor compared with its real size (scale bar, 5 μm). (D) Nano-scale “star” pattern was fabricated on the GST substrate by laser processing combined with microsphere. The corners of this “star” pattern were measured to be ~90 nm. It represented the imaging results of complicated patterns with arbitrary shapes (scale bar, 5 μm). (E) Optical image of a blue-ray disk target object. The 100-μm-thick protective layer was first removed before the imaging, revealing its key feature with 100 nm dimension. For the imaging, microspheres with 4.74 μm diameter were used in a reflection mode. The lines and grooves on the blue-ray disk could be clearly resolved in the virtual image (scale bar, 5 μm). (F) Imaging results of the microsphere installed in a confocal microscope setup. Ag quintuplet nano-dots on a glass substrate were fabricated as the target object for the microsphere imaging. The diameter of the nano-dot was 135.6 nm, and the gap between the nano-dots was 24.6 nm. Microsphere with a 5 μm diameter was placed on the sample for imaging. The inset at the top right corner shows the light intensity map of the areas marked by the white line (scale bar, 400 nm).

Microspheres provide the opportunity to directly face and overcome this challenge (Chen et al., 2019b). The discovery of microspheres' special light manipulation properties dated back to the year 2000 (Lu et al., 2000). In the experiment of laser cleaning, scientists were trying to remove the micro/nano-particles on a flat silicon wafer surface (Leiderer et al., 2000, Lu et al., 2000, Luk’yanchuk et al., 2000, Mosbacher et al., 2001, Munzer et al., 2001, Zheng et al., 2001). Many of these micro/nano-particles were of spherical shape, part of which was transparent. Laser at a high fluence (pulse energy per area J/cm^2^) was used to irradiate on these particles and remove them to clean the wafer. Unexpectedly, it was found that a number of tiny holes were formed on the wafer surface after the cleaning (Figure 1A) (Luk'yanchuk et al., 2002). These holes located directly under the spherical particles. The diameters varied for spherical particles of different sizes. For laser cleaning, it was disappointing as these holes were considered as damages to the wafer surfaces. To prevent it, efforts were devoted to study the mechanisms of the generation of holes (Mosbacher et al., 2001). These studies found that the size of the holes could sometimes be smaller than 100 nm, which can break the diffraction limit. They located in the regions that corresponded to the center beneath the spherical particles. Theoretical analyses showed that these spherical particles could focus the light into a region with sizes much smaller than the diffraction limit. Owing to its high intensity, the focused light ablates the silicon surface and generates these holes. Details are presented in the literature (Davim, 2013, Gao et al., 2015, Gramotnev and Bozhevolnyi, 2010, Guo et al., 2013, Luk’yanchuk et al., 2000, Maier and Atwater, 2005, Zheng et al., 2001, Huang et al., 2002).

Microspheres were soon used as a powerful surface patterning tool to improve the resolution from micro- to nano-scale by laser (Hong et al., 2006, Khan et al., 2011a, Khan et al., 2010, Liu et al., 2006, Okano et al., 2002). Remarkable results were achieved by off-axis irradiation, optical tweezers trapping, and micro-lens array (Figure 1B) (Daly, 2001, Hong et al., 2006, Khan et al., 2011b, Li et al., 2009, Lin et al., 2006, McLeod and Arnold, 2009, McLeod and Arnold, 2008, Piglmayer et al., 2002, Yang et al., 2005). Fundamentally, light can travel in the reverse direction along its optical path. Advances in nano-patterning inspired scientists to explore the performance of microspheres for nano-imaging. In the pioneering work, microspheres were directly deposited on the imaging sample with nano-structures (Wang et al., 2011). It was found that the microsphere produced a virtual image. The smallest feature size resolved went down to ~50 nm, far below the diffraction limit (Figure 1C) (Wang et al., 2011). This result was achieved without any sample pre-treatment or image processing. It served as the first experimental discovery that the diffraction limit could be directly overcome in an optical microscope. Many scientists repeated this experiment and further explored the functions of microspheres in different imaging conditions. Although microsphere provided new opportunities, it was found that using a microsphere to directly contact the sample surface was limited in many aspects as an imaging tool. Further development was proposed to lift the microsphere above the sample surface, which enabled a scanning mode. This optimization promoted this imaging method to have a similar operation process of conventional optical microscopes. The resolution in such non-contact mode was further improved to ~23 nm, similar to some table-top SEM systems (Chen et al., 2018a). These achievements soon drew intensive attentions of the primary industry players in the market. Co-development was arranged to further commercialize this technology. Existing engineering challenges of product development were solidly overcome by joint efforts, and the products ready for mass production were globally launched in early 2020. This technology was named optical microsphere nanoscope (OMN).

### Knowledge Hierarchy, Methodology, and Opportunities

The story of microsphere is a typical representative for the entire lifespan from scientific research to industrial products. This perspective aims to provide useful information, including a brief knowledge hierarchy in this field, commercialization methodology based on our past experience, and future opportunities for both OMN research and commercialization. Specifically, research works are first summarized to provide the essential knowledge of OMN. The methodology for commercialization is presented as a reference for the technology entrepreneurs to roadmap their projects. Finally, future research trends, application scopes, and opportunities are discussed. In this perspective, the first section summarizes the key research milestones about OMN. Both the contact and non-contact modes are introduced. Experimental setups and corresponding results are summarized. It aims to provide the timeline information for the development of this technology. Key points are highlighted, which are useful guidelines to design future research topics. By this summary of research progress, the key features and experimental requirements for OMN become much more familiar. The following part discusses the theories proposed for OMN, providing insights into the imaging process. The frontier research topics and future directions are also discussed from a scientific perspective. The second section focuses on the technology implementation, commercialization, applications, and future opportunities. The first part of this section introduces the process of commercialization for OMN from the technical perspective. Our commercialization process and strategies are summarized with key factors being highlighted. Suggestions are provided for other colleagues who are interested in technology transfer and commercialization. The next part primarily focuses on the new opportunities in various applications. Comparisons are made between OMN and other available commercial techniques. The future development of OMN is also discussed. For the audience with diverse backgrounds, this perspective does not touch too many theoretical analyses and derivations. The details and formulas can be found in the literatures cited in the corresponding sections.

## Section 1: Fundamental Researches in Microsphere Nanoimaging

Even with these remarkable progresses, OMN researches are still at its early stages with great potentials. This section summarizes the key researches with the future directions discussed. It contains four parts. We shall start with the experiment for microsphere in the contact mode. It was developed during the early stages of OMN research. The experiment for microsphere in the non-contact mode is then presented, which covers the development until the latest status ready for commercialization. These two parts introduce the basic concepts, imaging system designs, and the functionalities. With such understanding of microsphere nano-imaging, we present the available theories commonly used in this field. In the end, OMN is benchmarked with other optical imaging techniques to highlight its key features.

### Microsphere in Contact Mode

In most pioneering researches, there was lack of effective controls on the microspheres. Microspheres were directly deposited on the surfaces of the imaging samples, which were working in the contact mode. Experimental demonstration was reported in 2011, which was the first imaging result for microspheres (Wang et al., 2011). White light illumination was provided by a halogen lamp, and a 50 nm resolution on arbitrary patterns was achieved in this condition. The imaging process was conducted in ambient air. Diameters of the microspheres ranged from 2 to 9 μm. A conventional optical microscope was used to capture the image formed by the microsphere. In this microscope, 80× objective lens with numerical aperture (NA) = 0.9 was used. Samples of different materials and shapes were tested. The first experiment was conducted on metallic gratings. These gratings had 130 nm spacing and 360 nm line width. Microspheres with 4.74 μm diameter were directly deposited on top of the gratings. It was discovered that an image could be observed 2.5 μm beneath the sample plane, which was confirmed to be a virtual image. Such a virtual image was magnified by 4.17 times compared with the original sample. To further the investigation, the second experiment was conducted on fishnet gold-coated anodic aluminum oxide (AAO) with both 50 nm diameter pore size and 50 nm spacing. The experimental conditions were the same as the previous trial. The magnification of the virtual image was measured to be 8 times. Gratings and AAO were good examples to represent periodic lines and dots. Furthermore, samples of irregular shapes could also be observed. For example, a nanostar was fabricated and imaged (Figure 1D). Such complex patterns contained sharp corners of 90 nm size, which were directly fabricated on a DVD disk. Industrial samples, such as the blue-ray disk with 200 nm line width and 100 nm spacing, could also be clearly imaged (Figure 1E) (Wang et al., 2011). Furthermore, an imaging experiment was conducted on an array of microspheres of a hexagonal shape, which were made by the chemical self-assembly method. It was discovered that the virtual image formed by the microspheres could be stitched together to form an image with an extended field of view. A microsphere array can be used to further extend the field of view in OMN, as each microsphere serves as an independent micro-lens and provides the image at different spots across the surface. For the imaging target with a flat surface, a microsphere array can be an effective solution. For target with a curved or rough surface, additional measures need to be taken to overcome the challenge of focusing. These imaging results in air were solid evidence that the images generated by the microspheres clearly broke the limit predicted by the diffraction theory. It opened the gate to a new research direction of OMN, which challenged the resolution boundary of imaging with a purely optical approach. In these research works, critical knowledge was discovered through experiments, which are as follows: (1) a virtual image is formed by the microsphere beneath the sample surface, (2) such virtual image is magnified between 3 and 8 times compared with the original sample size, and (3) in the virtual images, tiny features with sizes smaller than the diffraction limit, can also be observed. After the first report of its observation power, hundreds of research papers followed to confirm these experimental results. These three important features prove to be the key factors in the OMN nano-imaging that requires theoretical description and quantitative modeling.

OMN preserves the advantages of the conventional optical microscopes. One flourishing research direction is to integrate OMN flexibly with other existing characterization tools to offer innovative functions or improve their performance. Three examples are presented, including confocal microscopes, Raman spectroscopes, and holographic microscopes. Conventional confocal microscope uses a focused laser beam to scan through the sample surface. The focused laser spot is produced by an objective lens, and signals are collected in an optical microscope via a pinhole. 3D imaging is available by stacking the information collected in a layer-by-layer method. One silica microsphere was placed before an objective lens in the confocal microscope. This microsphere further enhanced the focusing of the incident laser. It also reduced the noises from the side lobe (Yan et al., 2014). In air, samples with feature sizes of ~25 nm could be clearly resolved (Figure 1F). In this case, the best resolution was around one-seventeenth of the central wavelength of the illumination. Raman spectroscope is widely used to determine vibrational modes of molecules and characterize the chemical species. Coherent anti-Stokes Raman scattering (CARS) microscope is a commonly used instrument. As CARS is a nonlinear optical process, signals generated are weak. Microspheres were used to enhance signal generation as well as collection for CARS (Huang et al., 2014, Zheng et al., 2015). Microspheres were deposited on sample surfaces. A pulsed laser was focused by a 25× objective lens on the microspheres. Microspheres further focused the incident laser to provide an enhanced pumping light intensity. The signal was then collected by an optical condenser with a band-pass filter. Compared with the reference sample, this setup provided a 12.5 times enhancement. This result was highly promising for CARS researches. With such an enhancement, the intensity of the pumping laser can be greatly reduced to provide similar results as from the conventional design. A reduced pumping light intensity prevents the risk of optical damages and extends application scopes for CARS. Based on this example, it can be seen that OMN has great potentials to be combined with the spectrum analysis. Compared with the electron-based microscopy, optical signals contain abundant information in its spectrum. Combined with the state-of-art Raman technologies, OMN can also detect the materials composition. The enhanced electromagnetic fields at the focal point of the microsphere can further improve the Raman signals. More opportunities can be further explored, including the vivid characterization of bio-structures in the living cells and study of chemical reactions dynamically in liquid environments. Being at an early stage, the current studies primarily focus on a straightforward addition of microsphere in one spectrum detection device. Many more topics can be considered. Microsphere can be used to design innovative substrate for surface-enhanced Raman spectroscopy, which fully utilizes its light manipulation properties. Considering its small size, microsphere can be installed at the tip of optical fibers, which provide additional capabilities to characterize both the morphology and spectra inside living creatures' bodies.

A digital holographic microscope can also be upgraded by implementing microsphere (Aakhte et al., 2017). In this experiment, a microsphere with a relatively large diameter (529 μm) was placed between the Mirau microscope objective lens and the sample. Digital holograms were formed by recording the interference patterns of the reference beams and object. The reference and the signal beams are reconstructed to numerically calculate the image of the samples. It was found that the microsphere can improve the magnification by ~5.5 times. This setup was used for biological applications, such as cell identification. With the help of the microsphere, it could distinguish healthy and unhealthy cells for thalassemia minor.

In most of these pioneering works of microsphere researches, there was no effective approach to control the microspheres. Hence, most of the experiments were conducted in the contact mode. Contact approach is helpful for the understanding of imaging mechanisms. However, it is dramatically limited for its practical applications due to several challenges. First, the microsphere has a restricted field of view. A deposited microsphere can only provide a very limited range of sight. The position is restricted to the area where the microsphere resides. It lacks the flexibility for a general-purpose imaging tool. For most of the experiments, deposition of the microsphere is a rather random process, highly challenging to be accurately controlled. When the microsphere is not on the target spot of interests, desired images cannot be obtained. Second, microsphere can also be considered as contaminants for many samples. One microsphere is required to be deposited on one imaging spot. It is also challenging to form a uniform large-area microsphere array on the sample surface. Pre-treatment and sample cleaning make it inefficient for many applications. To overcome these limitations, it is required to move the microsphere accurately in the non-contact method.

### Microsphere Working in Non-contact Mode

Owing to the limitations discussed in the previous section, it is required to develop a non-contact imaging mode. The key to achieve the non-contact mode is to develop a mechanism to accurately control the microsphere above the sample surface at a controllable distance, which enables fast scanning over the sample surfaces (Chen et al., 2018a). It requires mechanical holders to be fabricated to install the microspheres and improve the imaging process. Methods were invented accordingly to lift the microsphere above the sample surface. There are two categories of the approaches to control the microsphere:

#### Tip-Based Non-contact Method

**Figure 2:**
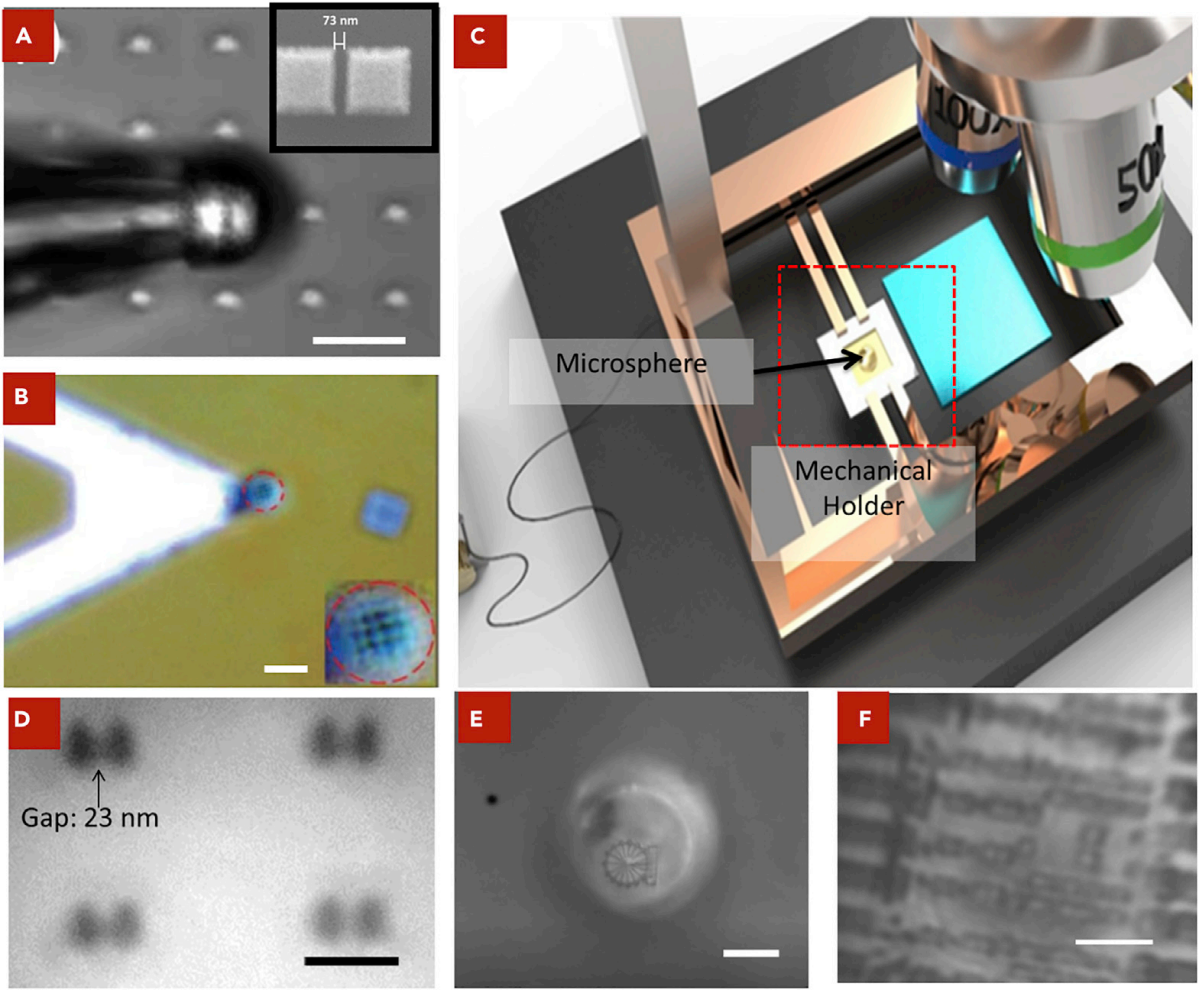
Optical Microsphere Nanoscope in Remote Mode (A) Imaging results for the non-contact made by a tapered glass rod. Sample is gold split-squares nano-structures imaged by microsphere in air. Microsphere has diameter of 6.1 μm (scale bar, 10 μm; inset: SEM image on the top-right showing the minimum feature size of 73 nm). (B) Imaging results of the microsphere hosted by an AFM tip to observe a nano-hole array fabricated on a Cr film. The dimensions are 130 and 170 nm, respectively, for the spacing and diameter of the holes. Microsphere has a diameter of 7.5 μm. Inset: virtual image of the nano-hole array by the microsphere (scale bar, 10 μm). (C) Design of the non-contact mode microsphere holder. One microsphere can be controlled by this mechanical holder to scan through the entire sample surface. A conventional optical microscope is then used to capture the magnified virtual image. (D–F) Imaging results for non-contact mode: (D) image of gapped 250-nm nano-dot pairs on a silicon wafer. The gaps between two nano-dots are 23–31 nm. Neighboring separated nano-dots are resolved through a 20-μm microsphere in oil immersion. Scale bar, 100 nm. (E) Imaging result of the “Singapore Flyer” by a 20-μm silica microsphere attached on an oil-immersion optical microscope. Scale bar, 10 μm. (F) Imaging results of the sample from a semiconductor production line with ~80 nm feature size; oil immersion under white light. Scale bar, 2.5 μm. (Microscope specifications for all three experiments: objective lens 100×, NA 1.4, and 405 nm laser illumination)

A microsphere has a relatively small size. Efforts were devoted to control it by attaching it on the top of a sharp tip, such as a tapered glass rod (Krivitsky et al., 2013). Ultraviolet glue was used to fix the microsphere. For a tip with a small diameter, numerical studies confirmed that the influence to the imaging was minimal. The diameter of the microsphere was 6 μm. In the imaging experiment, an objective lens with 50× magnification and 0.55 NA was selected, while light illumination was provided by a halogen lamp. The sample used for imaging has a feature size of 73 nm, coated with a gold film to improve the reflectivity. As demonstrated in Figure 2A, this sample can be clearly resolved. Compared with the contact mode, this early non-contact mode demonstrated some advantages. The microsphere could scan through the surface and move freely to the spots of interest.

Besides the tapered glass rod, other objects can also be considered to make a tip-based holder, including atomic force microscope (AFM) tip, needle, or even human hair (Chen et al., 2016, Duocastella et al., 2017, Wang et al., 2015). AFM system provides some privileges as a sample holder, as AFM platform has the entire system with the mechanical control at high accuracy. An AFM cantilever can be used to attach a microsphere with the help of optical glue. AFM cantilever has a thickness of 3–4 μm. In the experiment, it was used to hold the microsphere with a diameter of 7.5 μm. It is important to control the size of the tip similar or smaller than the diameter of the microsphere. Otherwise, the microsphere might not be able to approach the sample surface at a close distance. For imaging, the AFM cantilever was tilted by ~15° and a sample with a feature size of 130 nm can be clearly resolved (Figure 2B).

Tip based non-contact imaging method was useful at the early stage of OMN researches. It was obviously straightforward to achieve the setup. Its control mechanism only required a mechanical stage with high accuracy. The material of the tip was critical. Some researchers chose materials with high hardness and stiffness. This made the tip rigid and easy to be controlled. However, a rigid tip might be likely to break if it touched the sample surface accidentally. As the microsphere was attached to the tip by the optical glue, the microsphere was also likely to detach in this scenario. To further improve it, soft materials were considered. Soft materials offered damping protection when the tip touched the sample surfaces. The primary limitation of soft materials was that it made the tip vulnerable to vibrations. OMN imaging process required high accuracy of control. Vibration could influence the distance between the microsphere and sample, which made the imaging process not reliable. For these reasons, the tip-based method remains to be useful mostly for the purpose of scientific researches. It was more suitable for researchers to carry out proof-of-concept explorations. As the distance between the sample and the microsphere was relatively small, it was likely that an accident could happen due to vibration or operation mistakes as well as samples' roughness. For mass production, disruptive innovation is required to find a feasible way to hold the microsphere in a more reliable manner.

#### Membrane-Based Non-contact Method

Membrane-based holders provide a more robust solution to control the microsphere. In the pioneering studies, microspheres were fixed on a transparent glass strip by van der Waals forces (Chen et al., 2016). The process to prepare such a holder is simple and straightforward. Microspheres were first dispersed in deionized water. Both the microspheres and water were clean to prevent contamination in the following steps. Such dispersion was dropped on the glass strip, followed by heating for water evaporation. If the density of the microsphere in the dispersion was low, mono-layer of microspheres would attach to the glass strip after heating. Such a method could be used to make holders for the microspheres with relatively small diameters, normally smaller than 10 μm. A similar method was further developed to seal microspheres in a transparent film (Wang et al., 2018). For both methods, the deposition of microsphere was a random process without accurate control. The transparent film and glass strip both introduced aberration in the imaging process. To overcome these limitations, an advanced holder needed to be designed to provide a more predictable control and fix the microsphere at specific positions.

Hence, a mechanical system with high-accuracy control was proposed to fulfill these requirements (Chen et al., 2016, Chen et al., 2018a). The holder was made of steel, and the schematic of such a setup is presented in Figure 2C. Fabrication of this holder required advanced manufacturing tools of high accuracy. The principle was to drill a hole with size slightly smaller than the diameter of the microsphere. The microsphere selected in this experiment had 20 μm diameter. Special tools were then used to load and fix the microsphere onto the hole. The entire adaptor was fixed on a mechanical stage with accuracy down to 1 nm. Such high accuracy is required for the precise control of the microsphere in the imaging process. The entire system was put on an optical table with a proper damping system for the stability (Chen et al., 2018a, Hong et al., 2016). A conventional optical microscope was used to capture the virtual image produced by the microsphere. For the oil immersion condition, a 100× objective lens with NA = 1.4 was used. This system was tested on a sample fabricated by focused ion beam. The sample contained an array of paired nano-dots, whose separation was designed to range from 23 to 32 nm. It was clear that all the paired nano-dots could be clearly distinguished (Figure 2D). Besides periodic patterns, more complex arbitrary patterns were also tested. A “Singapore Flyer” was imaged and could be clear resolved (Figure 2E). The feature size in this fabricated pattern was ~50 nm. A sample directly from a semiconductor production line was also characterized (Figure 2F). An integrated circuit with 80 nm feature size can be clearly observed. All the imaging results presented are raw image data without any post-image processing. It was highlighted that an accurate control of the microsphere was critical to achieve imaging of the 23-nm feature sizes. The selection of the optical system in the conventional microsphere could also influence the imaging results. Generally, a large NA would yield better performance. Microspheres with different diameters have different fields of views. Those with smaller diameters provide a narrow field of view but better resolution.

Another key parameter for non-contact OMN is the distance between the microsphere and sample surface (Chen et al., 2019b). It remains a challenge to directly measure this parameter. Alternatively, an imaging experiment was conducted with a transparent film coated between the sample and the microsphere. The thickness of this transparent film could be tuned (Ye et al., 2013). In this experiment, the smallest feature size was 200 nm. The sample was an optical disk. In the sample preparation, a layer of transparent photoresist (SU-8) was spin-coated on the optical disk. Parameters in the spin-coating could be controlled to make SU-8 film at various thicknesses. Microspheres with 4.67 μm diameter were chosen. These microspheres were directly deposited on the SU-8 and imaged by a microscope with an objective lens of 100× magnification and NA = 0.9. To improve the light transmission from the SU-8 layer to the microsphere, ethanol was dropped on the surface. It was reported that such a measure could enhance the contrast in the imaging. White light illumination was provided by a halogen lamp. The central wavelength was 540 nm. It could be concluded that all the nano-grooves were resolved in the imaging process. However, when the distance between the microsphere and sample increased, the contrast decreased and the image became “blur.” In this experiment, it was not convincing whether this system was fully optimized. In the environment of high refractive index material, such as SU-8, the Rayleigh resolution limit was calculated to be 178 nm. However, the smallest feature size of the samples was 200 nm. Other experiments for the non-contact mode also reported super-resolution results (Chen et al., 2018a, Wang et al., 2015, Yang et al., 2016). In these works, the optimized distance between the microsphere and sample was reported to be within its focal length. For the microsphere with a diameter of 20 μm, this region is between the near field and far field. For the microspheres with a large diameter of 400 μm, this region is in the far field and the system lost its capability of super-resolution (Yang et al., 2016). It was also reported that the specifications of the camera in the conventional optical microscope also affected the final imaging results.

The methods to accurately control the microsphere gradually developed from the contact method to the non-contact method, from tip-based mode to membrane-based mode (Chen et al., 2019b). Every method has its unique features, pros and cons. Contact methods are straightforward and simple for the research purposes (Luk’yanchuk et al., 2017, Pena et al., 2010). They do not require a sophisticated setup to provide the nano-scale movement required to fix and control the position of microspheres. Their functions are sufficient for many proof-of-principle experiments but limit the capability to flexibly observe the point of interests on one sample. Tip-based method is the middle stage between the contact method and membrane-based method. The tip-based method provides a basic level of control of the microsphere. Owing to the mechanical strength of the tiny tip holders, their reliabilities and stabilities are limited. Membrane-based methods are the current state-of-art approach to control the microsphere. Membrane-based methods overcome the limitations of contact and tip-based methods. They provide the accuracy, reliability, and stability required for the microsphere imaging. Devices based on membrane-based methods can demonstrate similar functions and operation as the conventional optical microscopes. It took a long time and great efforts to design and master the fabrication of the membrane-based method. The primary weakness is that the membrane-based method is rather complicated and challenging to be set up without related technical experience.

### Theories of Microsphere Nano-Imaging

Since its experimental discovery, various approaches were developed to theoretically explain the microsphere imaging. In such a process, there are three key components: (1) imaging sample of interest, (2) microsphere, and (3) conventional optical microscope imaging system. Experimentally, it was found that the function of the microsphere was to form a virtual image. Hence, the first two components can be considered together. Based on it, the imaging process can be divided into two steps: (1) the microsphere manipulates the light from the sample and forms a virtual image and (2) conventional optical microscope captures this virtual image. The second step is a process with well-established theories. As a result, the key to the theoretical studies mostly focuses on the first step, which is the light manipulation of the microspheres in a virtual image formation. In the following section, several theoretical approaches are reviewed. As a perspective for a general scope, it will not cover detailed theoretical derivations. More information can be found in the literatures (Chen et al., 2019b).

#### Virtual Image Theory

In nearly all the imaging experimental processes, a virtual image is observed beneath the sample surface (Wang et al., 2011). Virtual image theory is proposed to model microsphere's functions in this process (Chen et al., 2018a). It focuses on microsphere's light magnification and studies the key parameters that affect the virtual image. Virtual image theory applies the knowledge of a spherical lens, which can produce similar virtual images. Both the microsphere and the spherical lens have similar geometrical shapes. The mechanisms from the spherical lens are modified to explain the virtual image formation from the microspheres (Guo et al., 2016). In this process, a microsphere first manipulates the electric fields from the sample surface. After its manipulation, the electric field emitting out from the microsphere is equivalent to the electric field emitting from a virtual image. Owing to this equivalence, this virtual image can be considered to replace both the sample and microsphere, which then becomes the target to be captured by the optical microscope system. This theory can explain all three key features observed in the experiment. First, the virtual image formation is consistent with the experimental observation. Second, the magnification can also be understood similarly to the theories of spherical lens. Third, due to the magnification, features that are smaller than the diffraction limit are magnified, which becomes larger than the diffraction limit in the virtual image. Hence, the conventional optical microscope can resolve these sub-diffraction features in a magnified virtual image. For example, it is challenging for a conventional optical microscope to image the features smaller than 200 nm. If the microsphere can provide a virtual image with a magnification of 4, a 100-nm feature in the sample becomes 400 nm in the virtual image. In this case, the key feature size is 100 nm (original sample) < 200 nm (diffraction limit threshold) < 400 nm (virtual image). The microsphere enables a conventional optical microscope to visualize tiny features beyond the diffraction limit.

Owing to the small size of the microspheres, its function is not exactly identical to the spherical lens. For the spherical lens, the derivation for the theoretical model is based on the geometrical optics in the far field. This derivation is valid when the size of the spherical lens is much larger than the wavelength of the incident light. For the microspheres, this assumption is not always valid. In most of the microsphere experiments, the microspheres are in close contact with the sample surfaces. Its distance is within the transition zone between the far field (10λ ≈ 5 μm) and near field (1/10λ ≈ 50 nm). Hence, a correction needs to be made on the formulas for spherical lens. In the virtual image theory, an additional effective modification factor *k* is introduced to account for the difference from the spherical lens. This factor depends on many parameters, such as the refractive index of the microsphere, the distance between the microsphere and sample, and the diameter of the microsphere. Numerical methods can be applied to predict *k* in different experimental conditions.

Generally, the virtual image theory considers both the intrinsic properties of the microsphere and the external factors of the environment. This theory is consistent with the simulations and experimental results observed in different conditions. It provides guidelines to optimize the performance of microspheres in various circumstances. However, this theory is also limited to certain perspectives. For example, in most experiments, effective control of the microspheres is lacking. It becomes challenging to estimate the distance between the sample and microsphere. Furthermore, it is also challenging to decide the exact form of the *k* factor. In addition, this theory is inefficient to interpret the super-resolution imaging using the microsphere, as it only explains the magnification without further revealing the details of how the microsphere can resolve the small features beyond the diffraction limit. There is lack of a detailed theory to explain how microspheres can reveal the details beyond the diffraction limit in the imaging process. Virtual image theory provides an accurate illumination to understand the imaging process of the microspheres. It still requires continuous theoretical development to understand the details of this process.

#### Other Related Theories

Other theories were also proposed to explain the mechanisms of the microsphere imaging. These theories include Mie theory and evanescent wave conversion (Duan et al., 2013, Lee et al., 2013, Wang et al., 2011, Yang et al., 2016). Mie theory is useful and widely applied to calculate the manipulation of light for a spherical object. Mie theory can be successfully applied to explain the light-focusing behavior of a microsphere. However, the OMN imaging process is different from a focusing process. Mie theory cannot predict the virtual image, magnification, and the enhanced resolution. It is useful for the understanding of the interaction between microsphere and incident light but requires additional development before it can be used to describe an imaging process. Furthermore, evanescent wave conversion theory is used to explain the enhanced resolution of a microsphere imaging. For the conventional optical microscope, only the information in far field can transmit and reach the sensor. The information in the evanescent wave is lost. In this theory, microspheres close to the sample surface can convert some evanescent waves into propagating waves due to the scattering of microspheres. As some evanescent waves can be converted and then detected by the conventional optical microscope system, information of higher spatial frequencies can be captured, which is equivalent to the detection of more detailed information from the sample surfaces. However, this theory also requires additional development. A number of researches report the experimental observation of super-resolution features when the microsphere is working in a non-contact imaging mode. It is beyond the scope of this theoretical explanation. Furthermore, this theory cannot explain the virtual image and magnification and requires further investigation.

### Benchmarking with Other Optical Nano-imaging Technologies

Imaging of the nano-scale objects is critical for many researches as well as applications. Many tools have been invented, and some of them are awarded Nobel prizes. In this perspective, we compare two primary categories of nano-imaging tools (Hao et al., 2013). Generally speaking, nano-scale imaging tools can be categorized as (1) vacuum-based type, and (2) non-vacuum-based type. The vacuum-based type relies on the electrons. Examples are scanning electron microscopes and tunneling electron microscopes. Vacuum-based tools can offer extremely high spatial resolution to atomic level. From an application perspective, their functionalities and flexibilities are limited by the requirement of vacuum conditions. For many biological and chemical substances, special sample preparation methods need to be adopted. The cost factor can also be one concern in mass production applications.

**Figure 3:**
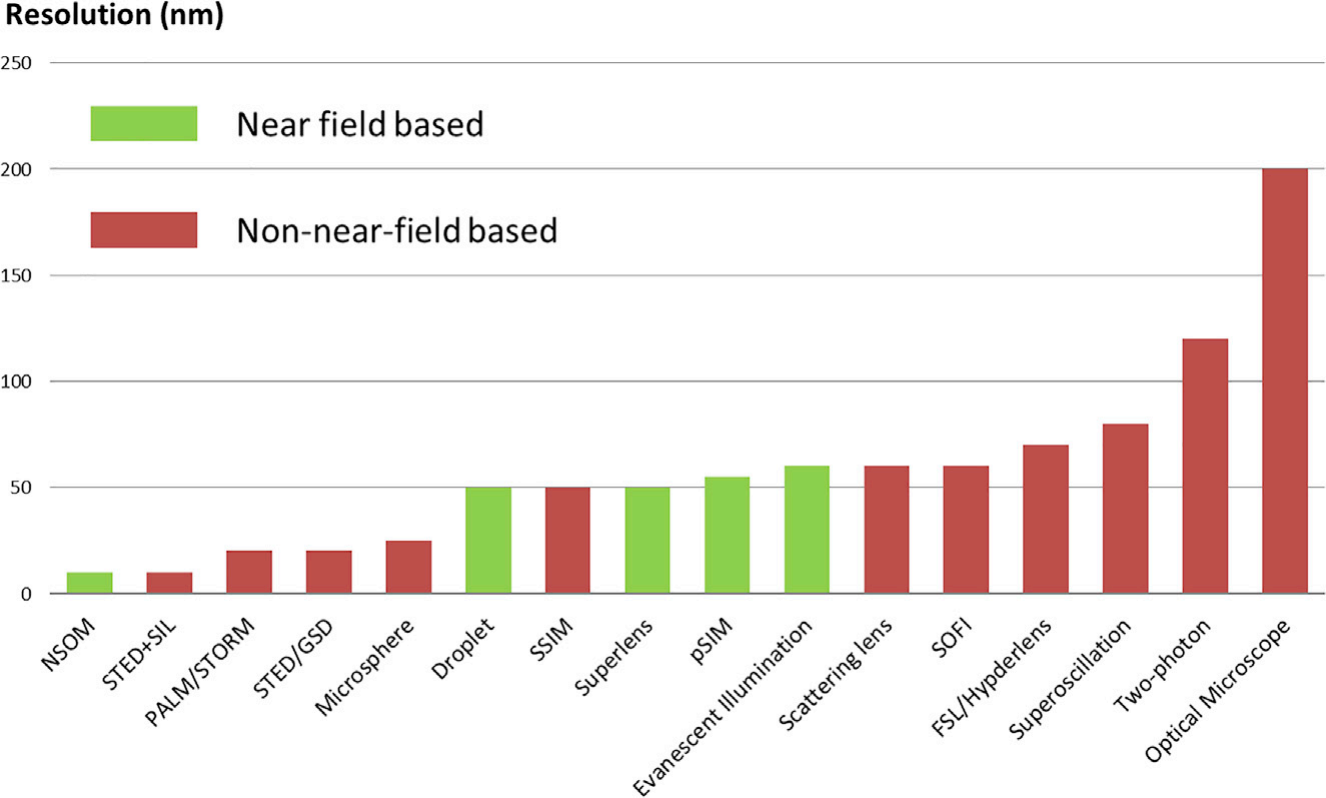
Summary of the Imaging Performance for Common Optical Imaging Tools Green color denotes near-field based tools, and red color denotes non-near-field-based tools. **Droplet:** Droplet lens detection method **Evanescent illumination:** Evanescent illumination detection method **Hyperlens**: Hyperlens detection method **Microsphere:** Microsphere-based detection method **NSOM:** Near-field scanning optical microscope **Optical Microscope:** Conventional optical microscope **PALM**: Photoactivated localization microscope **pSIM:** Prototyped structured illumination microscope **Scattering lens**: Scattering lens detection method **SOFI**: Super-resolution optical fluctuation imaging **SSIM**: Structured illumination microscopy with the nonlinear approach **STED**: Stimulated emission depletion microscope **STORM**: Stochastic optical reconstruction microscope **Superlens:** Superlens imaging method **Superoscillation**: Superoscillation detection method **Two-photon**: Two-photon microscope

For the non-vacuum-based tools, optical approaches are widely used. Optical approaches offer an abundant source of information via the analyses of light intensity, spectrum, phase, polarization, wavelength conversion, and orbital angular momentum. For the nano-imaging, the widely used optical tools are summarized in Figure 3. These imaging tools can be categorized based on two criteria: (1) resolution and (2) working distance. Conventional optical microscope is also included as a reference. Recently, hyperlens is also proposed as one option to achieve super-resolution nano-imaging (Kim and Rho, 2015). However, due to the challenges, such as fabrication and energy loss issues, the experimental results have not yet fully demonstrated the potentials predicted by the theory. Recent development of metamaterials for imaging purposes is still limited by the diffraction (Arbabi et al., 2018, Chen et al., 2018b, Kwon et al., 2020, Lee et al., 2018, Li et al., 2017, Lu and Liu, 2012, Tang et al., 2015). Resolution determines the minimum feature size observable, and working distance influences its functionality and flexibility. For those whose resolutions are worse than OMN, most of them still suffer from the diffraction limit. The application scope is restricted mostly to conventional areas. For the emerging needs, including the detection of virus or defect analyses in integrated circuit devices, such resolution is not sufficient. Photoactivated localization microscope (PALM), stochastic optical reconstruction microscope (STORM), structured illumination microscope (SIM), stimulated emission depletion microscope (STED), and near-field scanning optical microscope (NSOM), have superior resolutions compared with OMN. PALM, STORM, and STED were developed based on fluorescent microscopes. PALM and STORM rely on the characterization of single fluorescent molecules by the photo-activation process. In the operation process, the location of the fluorescent molecules can be calculated accurately and optical images can be reconstructed by scanning across the sample and mapping all the emission fluorescent molecules. STED has a different mechanism. STED achieves super-resolution by combining two laser beams together for the pumping of fluorescent molecules. One of the laser beams is used as the excitation, and the other is for depletion. When these two laser beams irradiate simultaneously, the area of illumination at the focal point is minimized, which enhances the resolution by the selective deactivation of fluorescent molecules. Compared with these florescent microscopes, OMN does not require any sample preparation, offering a ready-to-use imaging capability, especially appealing to samples that are challenging for fluorescent dyes. There is no photobleaching and low risk of sample damage. Also, no contamination is introduced. Furthermore, OMN is a highly flexible approach that operates in ambient air, water, and oil. It corresponds to a much wider application scope and can even be integrated with the fluorescent-based imaging tools to achieve improved results. Second, the working distance also influences the selection of imaging methods. NSOM is a representative based on near-field analyses. NSOM uses a specially designed optical tip to capture the near-field optical information from the sample surfaces. Such an optical tip then scans through the surface to characterize the surface optical parameters at different locations. This information is then mapped together to form an image. For SIM, several approaches apply the near-field illumination. They use patterned light to illuminate the sample and collect the information accordingly. Methods based on the near field are mostly restricted by the short working distances at ~10 nm. The device is designed to be in close contact with the sample. OMN has a relatively long working distance at a few microns. Its operation is much faster than the near-field methods based on a scanning approach. A long working distance also permits non-contact imaging, which allows detection without any influence to the sample being observed. For example, the little-energy-loss transparent microsphere for OMN can function in real-time imaging, providing nano-scale observation power to explore the key mechanisms of living biological systems and on-going chemical reactions. Such a fast, non-contact, and real-time imaging tool has great potentials to go beyond the laboratory and find intense field or industrial applications.

## Commercialization and Applications

### Technology Transfer

In the previous section, we first introduce the methodology for technology transfer. As a perspective, our related experience can provide helpful references for future technology commercialization. In general, our OMN project can be divided into two stages. The first stage is technology development. The result for this stage is a proof-of-concept demonstration protected by patents and other intellectual properties (IPs). The second stage is commercialization and spin-off. In this stage, we spin off a company and further push our technology ready for mass production.

#### Technology Development

The story of OMN is a good case study for high-technology commercialization. In the first part, we summarize the key milestones to share our experience. The key events for technology development in our research group are presented in the timeline below (Table 1).

**Table 1:** Intellectual Property Generation (2000–2017)

Time (Year)	Milestone
2000	Discovery in the laser cleaning experiment and exploration for laser nano-patterning.
	Pioneering work in the field of microsphere research. It made our research group one of the first batch of teams in the field of microsphere research. We started to communicate and connect with other teams internationally in the same research direction and accelerated the research progress.
2010	Early-stage research and first publication on the nano-imaging with microsphere.
	Pioneering work of OMN nano-imaging. We were among one of the few research teams in the world to explore microspheres for imaging and gained the knowledge to clearly understand the opportunities and challenges in the field, which finally guided us to propose the research project to build the non-contact OMN system.
2014 to 2017	Research project to design the non-contact control mechanism for microspheres. Our non-contact mode OMN system clearly observed samples with 23 nm feature size.
	This research project generated the key IPs for the OMN working in the non-contact mode. It was a huge step forward to overcome many technical limitations and make OMN ready for the next stage of mass production.

In this phase, we designed the laboratory-based experimental setup for microsphere imaging in the non-contact mode. International patents are filed with the assistance of the Industry Liaison Office of the National University of Singapore. OMN also won the national-level award for engineering innovation and represented Singapore to win the top award in the Association of Southeast Asian Nations. In the scientific community, the OMN has already become known to many groups and has been recognized for its capability to break the optical diffraction limit. Solid technology innovation and invention can be rather time consuming, especially for the bottleneck problems for critical areas. The technologies patented and directly related to the commercialization have mostly been invented between 2014 and 2017. However, the pioneering works dated back to 2000, which lay down the solid foundation for the innovations in the later period.

Technology innovation stands at the centre of the commercialization. There are a few key points to make the technology invention process productive. (1) Always being updated on the latest progresses of the research field as well as its most challenging tasks. This knowledge inspires innovation. In our process, it led us to use the knowledge in surface nano-patterning to solve the problems in nano-imaging. (2) Keeping good communications with other academic teams and industrial players. This helped us to further discover the value of our work and modify our R&D direction. (3) Fully cooperating and using the incubation platforms and commercialization channels. Professionals for technology transfer offered advices and suggestions to protect our technology with patents. We also worked with them to anchor the resources beyond the academic field. These measures kept us in the right direction through the journey to develop OMN, which reduced the number of mistakes and cost of exploration in the new adventure journey.

At the end of the technology development, a few factors are essential. (1) Solid knowledge of the inventions and its corresponding applications. This is a critical and basic requirement for the next stage of commercialization. (2) Patents and other IPs to ensure the technology is well protected. (3) A core technology team that is capable to build and further improve the prototype. (4) It is the best option to have reputations and supporting materials to prove the value of this technology to the audience beyond this field. Publications, awards, and news reports all contributed to this point. At the end of this stage, the technical readiness level should reach 6 or 7, where a model or prototype is ready for demonstration in a simulated operational environment and even for field testing in an operational environment.

#### Commercialization and Spin-off

**Figure 4:**
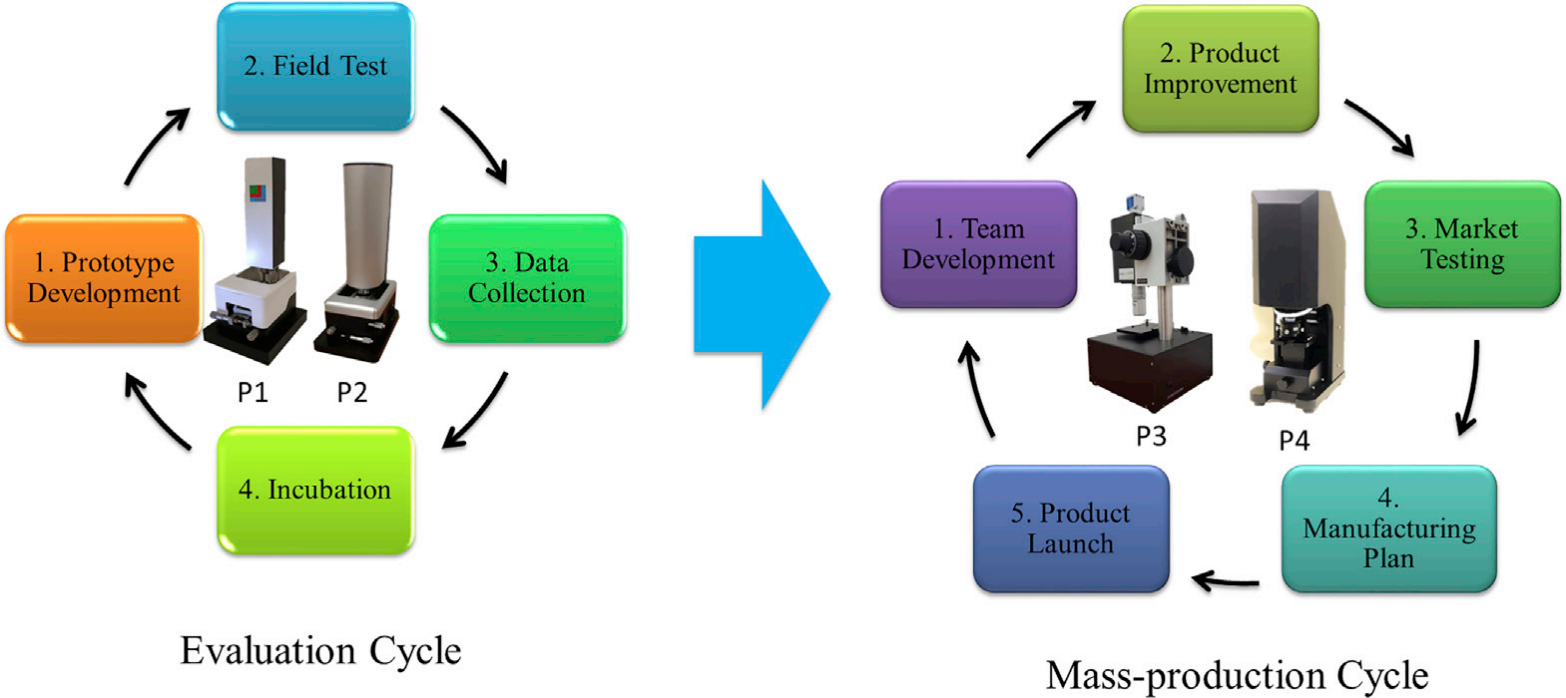
Strategy and Flow Chart for the Commercialization Process Key steps are summarized in the commercialization and spin-off phases. The entire process can be divided into two cycles: (1) Evaluation cycle and (2) Mass-production cycle.

Our strategy for commercialization can be summarized in the process as shown in Figure 4. It is a simplified work flow that only highlights the key steps. The entire process contains two cycles, namely, the Evaluation cycle and the Mass-production cycle. The Evaluation cycle requires limited resources and experiences a higher risk. The Mass-production cycle requires much more resources and aims at the launch of the product ready for the market. This two-cycle strategy can minimize risks and reduce resource consumption. If a project cannot survive the first cycle, time cost and efforts of the team can be used to develop new technologies.

In the Evaluation cycle, prototype development refers to the process to build the first prototype based on the IPs and proof-of-concept setups. The picture of our first functional prototype is shown in Figure 4, marked as **P1**. Prototype, which represents the near-desired configuration, does not need to be perfect. The key is to demonstrate the main functions. Field test is conducted to evaluate the prototype in a real market environment. The first field test should be selected carefully. The best partner is a team who has close relationships with the developers. Frequent communication is required to fully evaluate the knowledge collected in the previous laboratory environment. Prototypes can be loaned to field test partners and monitored closely to find out more information on how these prototypes can be improved to better help the partners. Data collection comes together with the analyses and plan making. The information and feedback received from the field test should be discussed within the team. Urgent issues regarding the primary functions need to be solved immediately. Minor issues regarding the incremental improvement can be solved in the next stage of development. Incubation is the last step. It includes the roadshows of the product in various entrepreneurs' events, cooperation with the professional incubation channels, and early discussion with potential investors, etc. The prototype needs to be evaluated at the end of this step: Whether is it ready for the next cycle? Does it need to be further improved until several key criteria can be met? Or should resources be kept for new ideas and developments due to some critical issues challenging to be solved in the short term. Rational discussion and evaluation are beneficial for everyone to make the right decision. **P2** in Figure 4 shows our prototype after this cycle. This Evaluation cycle requires mostly technical development, and the size of the team can be relatively small. Even part-time staff can contribute significantly.

After the completion of the Evaluation cycle, the prototype should already have potentials to approach the market. The next Mass-production cycle aims to build a small-scale business. This can be a journey with various challenges. Talents need to be recruited based on the information collected in the first cycle. The Chief Executive Officer is preferred to work full-time and put this start-up at the top of his priority. An expanded team can further push product development. The standards need to be raised after SWOT analyses. The aim is a product ready for mass production so user experience must be considered. It will be good to benchmark with other similar products in the market if available. The first product can be sold to a small group of early customers. It is a test-sale scenario, and standard customer service should be provided. Information is collected for the next step of strategizing manufacturing plan. At this stage, different partnerships need to be established. An evaluation needs to be conducted to check if all the elements are ready to kick start the business, from the collection of customer orders to the service and warranty of products. The final stage is the product launch. This marks the beginning of sales and business promotion. Potential distributors are developed to reach a broad scope of customers in different areas. **P3** and **P4** in Figure 4 show our products ready for mass production. These two types of products are developed based on different customers' requirements. In the Mass-production cycle, many more resources are required. The team involves more full-time staff to deal with different challenges. Our commercialization process is hardware-based products. Compared with the software start-ups, it has a more complicated process to manufacture the products and reach the customers. For this reason, the team requires a combination of experienced experts and motivated young talents. A solid understanding of the target area can minimize the chance of making mistakes in this process.

In practice, our team started with three part-time co-founders. We spent around 13 months to complete the evaluation cycle. It started with the pure knowledge and a few simple setups in the laboratory. Based on them, we first designed and fabricated a functional prototype with basic functions. Early users in the universities were first approached for the field tests. These users had access to various categories of samples for a better evaluation of the prototype. With the feedback, the prototype is continuously improved. We also fully explored the opportunities provided by the technology transfer department of the university, which exposed OMN to groups of interested partners and investors. With these connections and opportunities, further investment was injected and more professionals were recruited to expand both the technology development and business development. The company kept growing into the current stage.

#### Key Factors

As a perspective for technology entrepreneurs, it is helpful to break down the process and highlight the key factors useful for future evaluation and planning. Based on our experience, the following factors are listed, which have great influences on the success of the commercialization (Table 2). These key factors are helpful for the internal evaluation and risk assessment. It should be noted that the journey of commercialization can be described as the “leaky bucket theory”; all the elements are important. In the beginning, it is not necessary to have high standards for all evaluation factors. However, to succeed in the end, every team is suggested to consider it seriously and keep improving the weakness continuously. It should be highlighted that factors for success also highly depend on the situations and nature of technology. They should be considered in the context of different environments.

**Table 2:** Factors for Success

Key Factor	Evaluation
Technology	Technology is always the central part. Our technology has a few key features. It provides a solution to a bottleneck challenge that limits the development of a key field. It is well protected by our patents and other IPs. The research process is long and a lot of know-how ensures our competitors can hardly copy and reach the same level of understanding easily. Our technology does not have significant limitations. OMN is highly flexible with a broad application scope. All these aspects can be considered to evaluate a technology.
Team	A good team is the foundation for almost everything. The requirements for the team are different at different stages. Our team only consisted of three part-time co-founders to develop the first prototype. It expanded rapidly as the project progressed. At different stages, it is critical to understand the core duties and there must be talents to work on them. For the secondary duties, outsourcing can be considered to reduce the cost and time investment. The core team members also need to maintain connections to potential talents. Start-ups develop very fast; at the early stage, recruitment can be more efficient with ready-to-use contacts of several candidates.
Finance	In the Evaluation cycle, the focus is on technology development. Finance is mostly related to the burning rate of the fund. For the Mass-production stage, finance becomes more complicated and critical. Budget plan needs to be made to ensure the operation. This forecast can be done with professionals with experience. It is critical to ensure that the fund can support at least 5 to 8 months' operation. Otherwise, fund raising needs to be arranged immediately.
Operation	Different teams have various cultures. The key of operation is to ensure that tasks can be completed before the deadlines. High efficiency corresponds to less time and resources consumed to complete the task. At the early stage, it is highly suggested to keep a transparent environment with good communication. However, for a deep tech start-up, it is also critical to establish a secured information control to ensure the secrets do not leak to the competitors.
Market	For our market research, we focus on two points: (1) the size of the market and (2) how to access the market. Besides the market research reports and data, we found it was important to directly engage the players in the same market. The global market is huge with many players. Some of them can become partners and develop a win-win situation. Field study can also give detailed information on the customers. It is more direct to influence product design. Technology needs to be widely accepted by customers with different requirements.
Manufacturing	Technology decides the future potential, and manufacturing decides the real quality. A good manufacturing produces high-quality products in a reliable manner. For hardware start-ups, manufacturing is mostly challenging. We cooperate with the leading company in this field to ensure our product is of high quality. To build a high-standard manufacturing line requires huge amount of resources and solid experience. A good partner can be a feasible choice for most start-ups. Such a partnership needs to be robust to reduce risks.

### Potential Research and Application Areas

**Figure 5:**
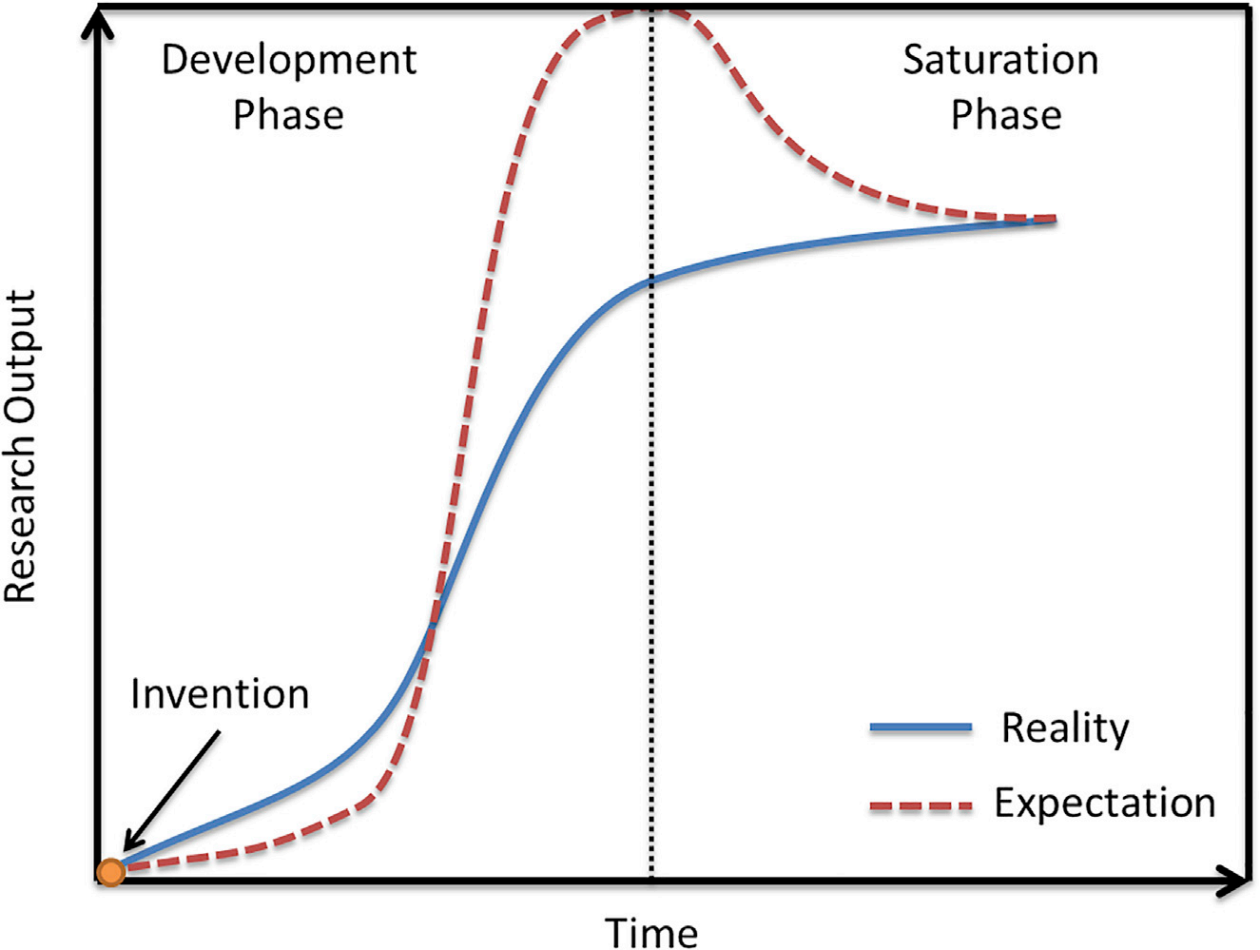
Research Output versus Time in Different Phases for a New Technology

The development of a new technology often follows the trend shown in Figure 5. In the development phase, the invention of a new technology is quickly applied in various fields, leading to a great number of new discoveries and a rapid increase of research outputs. The expectation of this technology also keeps increasing. Such rapid increase continues and attracts more and more scientists to follow the pioneering works and contribute to further developments. These efforts make this research field flourish and finally lead to more applications in industry. This was exactly what happened in the field of optical microscopes (Bradbury et al., 1968). From the invention to the great boost for many disciplines, optical microscopes eventually became one symbol to represent science. However, as the researches went deeper, the limitations were also revealed. The development of this field entered the saturation phase. The expectation drops as the boundary of the technology becomes more and more distinct. Finally, the research output saturates at a certain level, reaching its limit due to some bottleneck challenges. As discussed in the previous section, the primary bottleneck challenge is the resolution. To overcome it, it is required to improve the resolution and minimize the sacrifice to other key advantages for optical microscopes. OMN offers such a solution. OMN studies are still at an early stage. It is likely to enter into a rapid development phase when this technology is applied to more and more disciplines. In this section, we shall discuss these opportunities from three perspectives. The first one is further improvement of OMN and its integration with other imaging technologies. The second one is its potential applications for biological imaging. The third one is applications for advanced manufacturing.

#### Further Improvement of OMN

OMN is an imaging tool not fully optimized yet. Its potentials are still to be fully discovered (Chen et al., 2015, Chen et al., 2019a, Gao et al., 2017, Jin et al., 2016, Soh et al., 2016). The first promising research direction is to further improve this tool until it reaches the limit. The purpose of OMN is to produce virtualization of the nano-scale system. Its basic performance depends on several factors: (1) magnification, (2) observation power, (3) contrast, (4) aberration, and (5) working distance. All of these provide promising research directions. Magnification and observation power determine the smallest feature that can be imaged by OMN. Currently, the smallest feature size observed is ~23 nm. However, there lacks comprehensive theoretical study to model and predict the observation power of the OMN system with respect to the system parameters, especially for the limit of the smallest feature that OMN can image. Hence, it is important to ask the following: Whether 23 nm is the limit for OMN? What if we can further push the observation power down to 10 nm, which is sufficient to image the DNA? Or even to 5 nm for the characterization of the functional groups on the bio-membranes and defect detection in the most advanced integrated circuits? To approach its limit, one way is to further engineer the microsphere. For example, the focused ion beam can be used to fabricate structures on the surface of the microsphere, to manipulate its optical properties (Zhou et al., 2020a, Zhou et al., 2020b, Zhou et al., 2018). Similarly, other nano-scale fabrication tools can be considered to engineer the microsphere such as electron beam lithography. Furthermore, additional optical methods can also be applied to improve this system. For example, illumination is important for imaging. Special illumination patterns can be designed for microspheres. Hybrid methods can also be considered, such as a combination of the microsphere with micro-lens or other micro-scale optics (Gissibl et al., 2016). There is still plenty of new space to be explored and optimized regarding the better observation power.

Contrast, aberration, and working distance affect the quality of the image captured. Contrast is related to the signal-to-noise ratio of the image. In other words, it decides whether we can distinguish a pattern from its background. Aberration is a complicated topic. It decides whether the image is distorted. Working distance is also critical for many applications. Generally speaking, a long working distance is preferred. Working distance decides the gap between the microsphere and sample surface. For the imaging of transparent samples, it also influences the range of imaging beneath the sample surface. For conventional optical microscopes, a number of techniques are invented to improve these factors. Many of these techniques can also be applied for OMN. For example, phase contrast and differential interference contrast technologies can be applied for OMN's applications on biological samples (Arnison et al., 2004, Zernike, 1955). These two technologies apply the concept of interference to convert the phase information into amplitude information. Similarly, there are many methods used for the elimination of optical aberrations, which are used in lens and telescope designs (Guenther, 1990). Measures include (1) additional optical lens to correct the aberration; (2) computational methods and post-processing, and (3) other dynamic methods in adaptive optics and active optics. Working distance basically involves the geometrical design of the lens. One way is to engineer the microsphere to tune its working distance. It can also be controlled by changing the curvature of the micro-lens. Furthermore, adding extra components to form a compound lens system can also change the working distance.

In the previous section, we discussed how OMN can be integrated into the confocal microscopes, holographic microscopes, and CARS microscopes. In general, OMN can be considered as an advanced version of the conventional optical microscopes with almost no sacrifice of its advantages. Many technologies are based on conventional optical microscopes and OMN can be integrated into these systems. It is used extensively in microelectronics, nanophysics, biotechnology, pharmaceutical research, mineralogy, and microbiology (Bradbury et al., 1968). Most of these applications are compatible with OMN. Furthermore, unique opportunities can be introduced for the integration of OMN with spectrum analysis tools. For example, Raman spectroscopy is a highly sensitive technology capable of single-molecule detection. In practice, a sample often consists of complicated types of materials. This complexity introduces noises to the characterization as the signal of target materials can be easily interfered. OMN provides nano-scale virtualization, which helps select the areas of interests. It is able to filter out the noise and highlight the spectrum emitted from the selected area, which improves the accuracy of spectrum analyses. Combined with spectrum analyses, OMN can be further developed into a label-free characterization tool for non-invasive detection. In addition, image post-processing can also be an important research direction especially linked with big data and artificial intelligence (AI). The combination of optics and information technology creates values in both the conventional application scopes and emerging state-of-the-art research frontiers. Optical microscopes rely on human to process, analyze, and recognize the information in one image. Information technology can be used to enhance the capability of human by assisting the processing of images collected by OMN. For example, one image may be “blur” and full of aberration in human eyes. A trained AI model can dig out the useful information and characterize the object.

#### Biological Imaging

Conventional optical microscopes are used extensively to study animal and plant cells, bacteria, mitochondria, and nucleus, which range in size from hundreds of micrometers down to hundreds of nanometers. Viruses, ribosomes, proteins, lipids, and DNA have sizes between a few to tens of nanometers. Many of these samples are currently studied by the electron or fluorescent-based microscopes. Electron-based microscopes require the vacuum condition. Fluorescent-based microscopes require pre-treatment of the samples. OMN provides an alternative label-free and non-invasive approach. It can operate in air, water, or oil and does not need to pre-treat the samples. The ultimate goal for OMN will be a platform as flexible and universal as an optical microscope and offer the resolution like an ordinary electron-based microscope. It offers the method to study these biological substances in their living status, which can push our understanding on the mechanisms in biological systems into a new level (Vadivambal and Jayas, 2016). Its application to bio-imaging is wide. For example, efficient functioning of the human body involves a multilevel synthesis. They include many different structures and mechanisms from collective brain activity to the immune system. Bio-imaging is of great importance to study the immune, neuronal, or other cells, as well as the local redistribution and interactions of a relatively large set of proteins throughout one or different cells (Eggeling, 2018). This scope can be further extended to other animals, plants, and microorganisms. In this perspective, due to the limited contents, two examples are selected to show the potentials.

OMN can be used to image the virus. A properly optimized OMN can directly capture the morphology of bio-substances such as viruses of different shapes. OMN provides real-time analysis and the image can be captured instantly, which provide the capability to dynamically analyze the virus. The data captured in the imaging process can be further analyzed with the advanced analytical tools such as the state-of-the-art AI-assisted image recognition (Chen et al., 2019a). In virology, OMN is possible to record the entire process of infection with a living cell and monitor the change of the host cell in its different phases. It helps to understand the complex interactions of the infection process in detail. Researchers can then test and observe the effects of different vaccines at the fundamental level by directly observing the behavior of the cell. It can potentially greatly reduce the time required for the development of new medicines. Information can be collected from the single-cell level rather than recording the symptoms of the tested patients. OMN can also revolutionize the procedures and protocols to contain the spread of diseases. OMN is based on pure optical detection. It can detect the virus immediately without any usage of chemicals. If OMN can be widely deployed in the airports, railway stations, bus stops, and key junctions of transportation, it can provide the tests on all the passengers, and results are available almost immediately. OMN-based virus tests can be included in the public health security check process to screen out the infected passengers for further medical treatment. By placing it at the entrance of the building, it offers protection and makes the entire building a “green zone” free of suspicious cases. In this way, OMN can help to reduce the deaths, disclosers, and quarantine by containing the virus effectively.

The building blocks for DNA are nucleotides. Nucleotides have the width of tens of angstroms. A gene is made of hundreds to millions of nucleotides. If the observation power of the OMN can be further improved, or integrated with other bio-imaging techniques, to provide the visualization of several nucleotides, it is possible to directly engineer the DNA in a manner similar to a surgery. In this process, OMN can be used to scan through the entire DNA chain, locate the points of interest, and perform the manipulation required. With the proper database and assistance of AI, this entire process can even be fully automated.

#### Characterization for Advanced Manufacturing

Advanced manufacturing covers a wide range of industries. Characterization is critical for advanced manufacturing. One of these industries, the integrated circuit industry, has hundred billion dollars of market size and is continuously growing every year. High precision is the key for advanced manufacturing (Luo, 2019, Luo et al., 2019). Many mechanics, sensors, and information technologies are involved in achieving it. Quality control is important. Microscopes are widely used in manufacturing factories as a characterization tool for defects detection. Owing to the limitation of resolution, conventional optical microscopes can only be used for samples with micro-scale feature size. Electron-based microscopes are used for the quality control of samples with nano-scale feature size, such as the integrated circuits. However, as electron-based microscopes require the vacuum condition, only a small number of selected samples can be sent for examination. In addition, for non-conductive samples, metallic coatings are required. Samples characterized by this procedure can only be disposed afterward. OMN provides non-invasive and real-time imaging. With nano-scale resolution, it can be integrated directly into the production line. Every sample can be investigated by OMN. It can be combined with automatic image recognition, which is already widely used. Product failure of the standards can be picked out to maintain the best performance for every piece. It can save millions of dollars of economic loss due to product failure as well as the damage of the reputation due to the accidents. OMN can also be used to provide information in the installation of high-value systems. For many processes, the dynamic nano-scale monitoring provided by OMN ensures that components are installed at the proper positions during alignment.

Quality control and crack detection are not limited to the process of manufacturing. It is also essential to monitor the status of a structure. For example, OMN can be used to provide the characterization of key components in airplanes, spaceships, military vehicles, and plants of the oil industries. Metals are widely used materials for these industries. OMN can dynamically monitor their aging by analyzing metal surfaces and predict whether the structure is going to fail. For the welding points, OMN can also be used to characterize the quality. It can even be used in the food or chemistry production lines to monitor whether contaminants have already polluted the products.

## Conclusions

OMN is a highly flexible and universal technology. The opportunities are not limited to the applications covered in this perspective. This field is still at an early stage. New ideas and research directions shall emerge rapidly. The first section of this perspective helps to provide a short review to OMN. The key results and experimental designs are both covered to help researchers to understand this technology and design their own systems. The second section focuses more on the process of commercialization. The procedure and strategy of our team are shared as a good reference for future technology entrepreneurs. It often takes long for an innovative invention to develop into a product that is accepted by the customers. Challenges from various perspectives need to be overcome in this journey. After the milestone achievement of the OMN research, our motivation to start the commercialization is straightforward, as OMN can benefit a broad range of disciplines. Instead of the mode in academic cooperation, OMN is considered to be more easily accessible and beneficial to research groups and industry partners by making it commercially available. Although a lot of researches and efforts are still required to further develop OMN, more and more discoveries and innovations can be inspired through a wider range of extensive communication and cooperation.
